# Semaglutide in Cardiometabolic Diseases: SELECTing the Target Population

**DOI:** 10.3390/jcdd11050145

**Published:** 2024-05-07

**Authors:** Francesco Natale, Ettore Luisi, Rosa Franzese, Noemi Mollo, Achille Solimene, Valentina Maria Caso, Andrea Corvino, Paolo Golino, Giovanni Cimmino

**Affiliations:** 1 Vanvitelli Cardiology Unit, Monaldi Hospital, 80131 Naples, Italy; natalefrancesco@hotmail.com (F.N.); paolo.golino@unicampania.it (P.G.); 2 Department of Translational Medical Sciences, Section of Cardiology, University of Campania Luigi Vanvitelli, 80131 Naples, Italy; 3 Department of Precision Medicine, University of Campania Luigi Vanvitelli, 80131 Naples, Italy; 4 Pharmacy Unit, Monaldi Hospital, 80131 Naples, Italy; 5 Cardiology Unit, AOU Luigi Vanvitelli, 80138 Naples, Italy

**Keywords:** glucagon-like peptide-1 receptor agonists, cardiometabolic diseases, semaglutide

## Abstract

Cardiovascular diseases remain the main cause of death and disability worldwide. Despite the tremendous improvement in pharmacological, minimally invasive and rehabilitative strategies, global deaths due to cardiovascular diseases are still increasing. Additional risk factors have been recently proposed, and thanks to scientific progress, novel drugs for the control of the main risk factors focusing on the cardiometabolic pathways have been identified. Glucagon-like peptide-1 (GLP-1) receptor agonists represent an innovative step in the management of patients affected by type 2 diabetes mellitus. In addition to their significant efficacy on glycemic homeostasis, some members of this class of drugs have indications in the treatment of obesity. Furthermore, accumulated evidence in the literature has finally suggested a protective role in cardiovascular health. The possible role of GLP-1R agonist drugs (GLP-1RAs) on the mechanisms underlying chronic inflammation and the almost ubiquitous distribution of GLP-1 receptors could explain the enormous versatility of these drugs. Semaglutide is a GLP-1RA recently proven to be effective in cardiovascular outcomes. In the present article, we will review the available data on semaglutide in light of the most recent publications to better characterize the target population achieving cardiovascular benefits.

## 1. Introduction

Glucagon-like peptide-1 (GLP-1) is a hormone synthesized mainly in the distal section of the small intestine from L cells. Together with Gastric Inhibitory Peptide (GIP), it is one of the main mediators of the incretin effect and is responsible for insulin secretion in response to the ingestion of nutrients [1]. Specifically, through binding to its receptor (GLP-1R), GLP-1 stimulates insulin secretion and inhibits glucagon secretion, thus promoting the proliferation and differentiation of pancreatic beta cells by inhibiting their apoptotic processes [1]. It is also involved in determining satiety and slowing gastric emptying [2]. Considering their role in glycemic homeostasis, the benefits on body weight reduction and the recently shown protective effects on cardiovascular health, the use of GLP-1R agonist drugs (GLP-1RAs) is revolutionizing therapeutic approaches and not only for patients suffering from type 2 diabetes mellitus (T2DM) [3]. The current guidelines recommend this category’s drugs as second line in addition to metformin therapy, especially in patients with known atherosclerotic-related cardiovascular risk [4]. Within the cardiovascular system, GLP-1R is expressed not only by cardiomyocytes and endothelial cells but also in the autonomic nervous system. The biological pathways by which GLP-1 exerts greater protection against cardiovascular events are not completely defined yet. It is hypothesized that the reduction in post-ischemic damage, the regulation of lipid synthesis, the improvement of endothelial dysfunction and thickening medial intima are involved [5]. By reducing the production of inflammatory cytokines and reactive oxygen species (ROS), GLP-1RAs could mitigate the production of endothelial adhesion molecules, slow the formation of atherosclerotic plaques and inhibit the formation of the foam cells involved in the atherogenesis process [6,7,8]. The protective effects of GLP-1 could also be associated with the increased production of atrial natriuretic peptide (ANP) and nitric oxide (NO), resulting in a reduction in arterial pressure levels [9,10]. The relative increase in heart rate induced by GLP1RAs, reported by several works, does not appear to have any significant clinical implications in treated subjects [11]. Semaglutide is the latest GLP-1RA used to treat T2DM. Recently, it has been reported that in patients with pre-existing cardiovascular disease (CVD) and overweight/obesity but without diabetes, semaglutide significantly reduces the incidence of death from cardiovascular causes, nonfatal myocardial infarction or nonfatal stroke compared to placebos (the SELECT trial) [12]. In light of this recent publication, we will review the available data on semaglutide to try to define the legible candidate to obtain higher cardiovascular benefits from its administration.

## 2. Literature Sources and Search Strategy

We performed a non-systematic review of the literature by applying the search strategy in different electronic databases (MEDLINE, EMBASE, Cochrane Register of Controlled Trials and Web of Science). Original reports, meta-analyses and review articles in peer-reviewed journals up to March 2024 evaluating the clinical benefit of semaglutide in cardiometabolic patients have been searched. Glucagon-like peptide-1 receptor agonists, semaglutide, obesity, diabetes and cardiovascular risk were incorporated into the electronic databases for the search strategy. The references of all identified articles were reviewed to look for additional papers of interest to extrapolate the more recent available data on the link between semaglutide and its cardiovascular benefits.

## 3. Semaglutide: Molecular Pathways Underlying Clinical Benefits

Semaglutide is a GLP-1 analog with 94% sequence homology to native GLP-1 [13]. There are three key structural differences compared to GLP-1 that provide extended pharmacokinetics properties. The substitution of Ala with α-aminobutyric acid (Aib) at position 8 increases enzymatic (DPP4) stability because it protects against degradation [14]. The attachment of a linker and C18 di-acid chain at position 26 provides strong binding to albumin. The substitution of Lys with Arg at position 34 prevents C18-fatty-acid binding at the wrong site. Semaglutide has a half-life of 165 h in humans due, in part, to the non-covalent association with human serum albumin, which results in decreased renal clearance and protection from metabolic degradation [14,15]. Semaglutide selectively binds to and activates the GLP-1 receptor, the target of native GLP-1 [13,14]. Glucagon-like peptide-1 (GLP-1) is a major incretin hormone in humans that has multiple actions on glucose, mediated by GLP-1 receptors in the pancreas and the brain [16]. Semaglutide reduces blood glucose through increased insulin secretion and decreased glucagon secretion, both in a glucose-dependent manner. It also suppresses hepatic gluconeogenesis and reduces both fasting as well as postprandial glucose [17]. Semaglutide also causes weight loss, which is attributed to reduced energy intake, a delay in gastric emptying and reduced appetite [18,19]. GLP-1 receptors are also expressed in the heart, vasculature, immune system and kidneys. Semaglutide has a beneficial effect on plasma lipids, lowers systolic blood pressure and reduces HbA1c [20]. GLP-1R belongs to the receptor family coupled to the G protein [21]. At the pancreatic level, its interaction with GLP-1 determines the activation of the cAMP/PKA pathway [2,22]. Taking into account the ubiquitous expression of GLP-1R, it is not surprising that this gut hormone might have effects beyond glycemic control [21]. It has been reported that GLP-1 may exert an anti-inflammatory effect through different mechanisms acting via the down-regulation of cytokines, such as interleukin 1 (IL-1), interleukin 6 (IL-6) and tumor necrosis factor-alpha (TNF-α) [23,24,25], and the inhibition of the infiltration of inflammatory cells in the tissues [26]. Such phenomena appear to be directly mediated by the activation of GLP-1RA and not secondary only to the improvement of glycemic control in treated patients. It is well known that insulin resistance is a condition associated with chronic inflammation; thus, the anti-inflammatory effect could further explain the remarkable effectiveness of this class of drugs in terms of glyco-metabolic compensation and cardiovascular protection [26,27].

The data from preclinical studies do not indicate any specific risks for humans based on the conventional studies of pharmacology, repeated dose toxicity or genotoxicity [28]. In animal studies, semaglutide attenuates the development of atherosclerosis by preventing aortic plaque progression and reducing inflammation in the plaque [29]. It has been reported that semaglutide decreased levels of plasma markers of systemic inflammation in an acute model of lipopolysaccharide-related inflammation [6]. These data were further confirmed by the transcriptomic analysis of aortic atherosclerotic tissue, revealing that multiple inflammatory pathways were down-regulated [6]. In addition, semaglutide reduces activated macrophage activity in atherosclerotic lesions, reinforcing its anti-inflammatory properties [30]. Its role in carcinogenesis is still controversial. A study conducted on rats and mice over a 2-year period showed that semaglutide administration “at clinically relevant exposures” is associated with thyroid C-cell tumors [31]. The mechanism underlying rodent C-cell tumors is non-genotoxic and mediated by a specific GLP-1 receptor, which rodents are particularly sensitive to [31]. Continuous exposure to GLP-1R agonists up to 13 weeks was associated with marked increases in plasma calcitonin and the incidence of C-cell hyperplasia in wild-type mice [31]. However, real-world data [32], as well as systematic review and meta-analysis [33], do not support this correlation.

Some preclinical investigations have raised safety concerns about GLP-1RAs in eliciting pancreatic inflammation [34], cellular proliferation and pancreatic intraepithelial neoplasia [35]. While the clinical data seem to support the relationship between GLP-1RAs and pancreatitis [36], the association between semaglutide and pancreatic cancer or any type of cancer is not confirmed by a recent meta-analysis on 16,550 patients [33].

Since 2014, semaglutide has been the first drug approved by the FDA for chronic weight management, with clinical benefits reported by real-world data [37]. In animal models, it has been shown that semaglutide exerts these effects on food intake control and weight loss by modulating food preference and reducing food intake, thus leading to weight loss without decreasing energy expenditure in rodents [38]. This study showed that following an acute semaglutide injection, a marker of neuronal activity called c-Fos was activated in 10 brain areas, including those with GLP-1R, such as the hypothalamus and the brain stem, as well as in areas without GLP-1R, such as the central nucleus of the amygdala and the parabrachial nucleus [38] that are part of a non-GLP-1R-dependent neuronal network activated by the GLP-1R dependent areas [39,40]. This report indicates primary and secondary activation by semaglutide. These brain areas correspond to regions involved in appetite regulation related to meal termination [38]. Moreover, accumulating evidence supports the existence of GLP-1R-independent activation via GLP-1 degradation products that act through novel receptors or for passive transport through cellular membranes [41,42].

## 4. Semaglutide and Obesity: STEP by STEP for Weight Loss

In 2021 and 2022, the US and European regulatory agencies approved the use of semaglutide [43] at a dosage of 2.4 mg subcutaneously (sc) once a week for the treatment of overweight patients with at least one weight-related complication (such as hypertension, diabetes, dyslipidemia or sleep apnea) or obese patients, in combination with a low-calorie diet and regular physical activity [28,37,44]. As reported above, it reduces hunger pangs, increases satiety and slows gastric emptying, leading to an overall reduction in food intake and consequent weight loss [45,46]. The results of the Semaglutide Treatment Effect in People with Obesity (STEP) program trials, which aimed to evaluate the efficacy, safety and tolerability profile of 2.4 mg of semaglutide sc once a week in body weight management, were instrumental in the approval of this drug in this setting of patients [28]. In all trials of the STEP program involving non-diabetic adults, the population included males or females aged at least 18 years who were overweight (a body mass index (BMI) of at least 27) with at least one related weight comorbidity (hypertension, sleep apnea, diabetes or dyslipidemia) or obese (a BMI of at least 30), who had a history of, at least, one unsuccessful attempt at weight loss with a dietary regimen [47,48,49,50,51]. The population in the STEP 2 trial included adults with a BMI of at least 27 and glycated hemoglobin of 7–10%, with a diagnosis of type two diabetes made at least 180 days before screening for the trial [52]. Patients with a weight loss of more than 5 kg in the 90 days prior to screening, previous bariatric treatment for obesity, an estimated glomerular filtration rate (eGFR) <40 mL/min, and patients unable to adhere to low-calorie regimens or sustain regular physical activity, glycemia greater than or equal to 6.5 or a history of type 1 or type 2 diabetes or who had taken oral hypoglycemic drugs in the 90 days prior to screening (for trials in non-diabetic patients) were excluded [47,48,49,50,51,52]. In these trials, the drug treatment (2.4 mg of semaglutide sc once weekly versus a placebo or 2.4 mg of semaglutide sc once weekly versus 3 mg of liraglutide orally once daily) was combined with a hypocaloric diet of conventional food and a progressively increasing intensity of physical activity [47,49,50,51,52], while in STEP 3, it was combined with intensive behavioral therapy (IBT), where IBT is defined as 30 individual consultations with a dietician over the duration of the trial [48]. In this case, the treatment was administered together with a low-calorie diet of conventional food and regular physical activity, after an initial 8-week phase in which all patients were given a low-calorie diet of meal replacements [48].

Given the differences between Asian and non-Asian populations in body composition and, consequently, in the definition of obesity, the STEP program also set out to investigate the efficacy of semaglutide in this patient setting in the Asian population. To this end, the STEP 6 trial randomized 4:1:2:1 401 adults from East Asia (South Korea and Japan) who were obese (a BMI of at least 35) with at least one weight-related comorbidity or overweight (a BMI of at least 27) with at least two of these comorbidities (one of which had to be hypertension dyslipidemia or, in Japan only, diabetes), and diabetic or non-diabetic to 2.4 mg of semaglutide sc once a week or a placebo versus 1.7 mg of semaglutide sc once a week or a placebo [53]. The STEP 7 trial was also conducted for the same purpose. It considered a population of 375 predominantly Asian patients and randomized them 2:1 to 2.4 mg of semaglutide sc versus a placebo [54]. Each trial had a treatment period of 68 weeks except for STEP 5, where treatment lasted for two years [50], and STEP 7 lasted 44 weeks, plus a 7-week post-treatment follow-up period. The STEP 1 trial also included an extension phase in which some of the participants were followed up post-treatment, i.e., from week 68 until week 120 [55]. In all trials, semaglutide was well tolerated. The most common adverse events were gastrointestinal (vomiting, diarrhea, nausea, or constipation), which were generally transient and did not lead to treatment discontinuation. To reduce the incidence of adverse events, the dose of semaglutide was gradually increased over four weeks to a dosage of 2.4 mg [56]. Indeed, the incidence of these events was higher in the initial phases of drug titration [47,48,49,50,52]. The primary endpoint in all these trials was the change in body weight percentage at the end of treatment compared to baseline. In some of these studies, the percentage of subjects who achieved a weight loss of at least 5% from baseline at the end of treatment was defined as the co-primary endpoint. The secondary endpoints were the percentage of adults who achieved, at the end of treatment, a weight loss compared to the baseline of at least 10%, 15% or 20% and the change from the baseline in the cardiovascular risk factor values and obesity comorbidities (centimeters of waist circumference, blood pressure in mmHg and clinical outcomes assessed using individual scales). All trials achieved the primary endpoint, laying the groundwork for the approval of this drug as a therapy in body weight management in the USA and Europe. Trials comparing 2.4 mg of semaglutide sc once a week versus a placebo showed a significantly greater reduction in body weight in patients treated with 2.4 mg of semaglutide than in those treated with a placebo, with the estimated treatment differences ranging from −6.2% in the STEP 2 trial to peaks of −12.4% in STEP 1 and −12.6% in STEP 5 [47,48,50,52,53,54]. Regarding the co-primary endpoint, in all trials that considered it, a greater percentage of adults treated with semaglutide than those treated with a placebo achieved, at the end of treatment, a weight loss of at least 5% compared to at the time of randomization [46,47,49,51,52,53].

In further support of these important results, the STEP 8 trial, which compared head-to-head 2.4 mg of semaglutide sc once a week with 3 mg of oral liraglutide once a day (the first among GLP-1RAs to be approved in the treatment of obesity), also achieved the primary endpoint by showing significantly greater weight loss in the semaglutide-treated group, with an estimated treatment difference of −9.4%, and more patients treated with semaglutide achieving a weight loss of at least 10%, 15% and 20%, taken individually as secondary endpoints [51], thus demonstrating the superiority of semaglutide over liraglutide in body weight management. The STEP 4 trial, in which participants, after an initial 20-week period during which they all received 2.4 mg of semaglutide sc once a week, were randomized to continue treatment with 2.4 mg of semaglutide or to continue with a placebo until the end of treatment at 68 weeks, showed that switching to a placebo after a period of treatment with semaglutide resulted in a significant regain of some of the body weight lost while continuing treatment with semaglutide allowed weight loss to continue [49].

STEP 5 showed how the continuation of semaglutide treatment for a period of approximately two years leads to maintenance of the significant weight loss achieved in the initial phase (approx. 42 weeks) [50]. The extension phase of STEP 1 also showed that during the follow-up after the end of treatment at 68 weeks, there is progressive weight loss, additionally proving the loss of the positive effects on the cardiovascular risk factors during treatment [55]. These last three trials highlight an important limitation of this therapy, represented by the need to continue treatment to maintain the benefits obtained. This limitation, considering the chronicity of the pathology to be treated and the already existing shortage of the drug in question, as well as the fact that currently available trials have studied this in this setting for up to two years, could pose a problem in the management of patients starting this treatment.

The STEP 6 and 7 trials demonstrated a statistically greater change in body weight at the end of treatment compared to the baseline in patients in the semaglutide-treated arm, with an estimated treatment difference for 2.4 mg of semaglutide versus the placebo of −11.1% in STEP 6 [53] and −8.5% in STEP 7 [54], supporting the use of this drug for body weight control also in adults of Asian ethnicity.

In terms of secondary endpoints, it was interesting to note that in all trials, treatment with 2.4 mg of semaglutide sc once a week led to a significant improvement in the cardiovascular risk factors [57]. Indeed, a significant reduction in waist circumference centimeters was observed in patients treated with semaglutide in both diabetic and non-diabetic patients [51,53,54,57]. In trial 2, which exclusively involved diabetic patients, there was a reduction in glycated hemoglobin of less than 7% or even 6.5% in a significantly higher percentage of patients treated with 2.4 mg of semaglutide sc once a week than in those treated with 1 mg of semaglutide or a placebo [51,52,57]. Even in trials that considered non-diabetic patients, a statistically significant number of patients treated with 2.4 mg of semaglutide sc once a week, who at the time of randomization had a diagnosis of prediabetes (HbA1c ranging from 5.7 to 6.4%), switched to a normoglycemic state at the end of treatment [51,53,54,57]. Positive effects were also observed on arterial hypertension, showing a significant reduction in systolic blood pressure (PAS) and diastolic blood pressure (PAD) at the end of treatment in patients treated with semaglutide [50,52,53,55], with the exception of trials 2 and 4, which showed no statistically significant differences in PAD in the two groups [49,52,57]. The trial results show that semaglutide also improved the lipid metabolism profile of these patients, resulting in a significantly greater reduction in total cholesterol, triglycerides, low-density lipoprotein (LDL) cholesterol and very low-density lipoprotein (VLDL) cholesterol and a significant increase in high-density lipoprotein (HD) cholesterol in the semaglutide group compared to the placebo group [51,57], with the exception of the trials conducted in the Asian population, in particular STEP 7, which showed a statistically significant reduction only in total cholesterol, triglycerides and VLDL [54]. A clear pathophysiological explanation for this finding has not yet been provided, but taking into account the incretins’ mechanism of action, some hypotheses have been made. It has been reported that therapies that increase GLP-1 activity, including semaglutide, reduce postprandial and fasting levels of triglycerides and small dense lipoprotein cholesterol in association with a concomitant shift towards larger, less atherogenic particles (like HDL) [58]. This effect is supposed to be the consequence not only of the weight reduction achieved by using these drugs but precisely due to the direct action of GLP-1 both on peripheral receptors, where it would increase intestinal lipid absorption and hepatic production of triglyceride-rich lipoprotein [58] and on central receptors located in neurons of the nervous system and hypothalamus, where the activation of this signal pathway would lead to a postprandial reduction in triglycerides-rich lipoprotein apoB48 and triglycerides, as shown in preclinical studies [59].

A summary of the STEP program is provided in Table 1.

Given that the reduction in cardiovascular risk factors appears to be associated with a reduction in cardiovascular mortality, these results paved the way for a major trial (SELECT), whose aim was to assess the superiority of semaglutide over placebos in preventing major cardiovascular adverse events in obese or overweight non-diabetic patients [12].

To sum up, treatment with 2.4 mg of semaglutide sc once a week in obese patients proved to be a valid option for body weight management, leading not only to a significant reduction in body weight, even greater than that obtained with already approved drugs belonging to the same class but also to an improvement in weight-related comorbidities and cardiovascular risk factors for a period of time that was, unfortunately, limited to the duration of treatment, beyond which the benefits obtained appeared to be lost.

Lastly, the STEP program also included a trial (STEP TEENS) aimed at evaluating the possibility of using this drug in a population of 201 adolescents (aged 12 to 18 years) who were overweight (body weight above the 85th percentile), with at least one weight-related comorbidity, or obese (body weight above the 95th percentile), randomizing them 2:1 to 2.4 mg of semaglutide sc once a week versus a placebo for 68 weeks in association with lifestyle interventions. The results show, again, a significantly greater reduction in body mass index (BMI) and a significantly greater improvement in cardiometabolic risk factors in the semaglutide-treated arm than in the lifestyle-intervention-only arm, laying the groundwork for the use of this drug also in adolescent patient settings [60].

A recent randomized phase three study analyzed the use of oral semaglutide at a dose of 50 mg once daily in this same patient setting (obese or overweight non-diabetic adults with at least one related-weight comorbidity) compared with a placebo, demonstrating once again statistically greater weight loss in the semaglutide-treated arm but noting a high incidence (80%) of gastrointestinal adverse events [61]. This study could pave the way for the use of oral semaglutide in this patient setting, but we are currently still a long way from market approval.

## 5. Semaglutide and Prediabetes: The Way for Early Prevention

Prediabetes, also known as “intermediate hyperglycemia”, is a common condition defined by the American Diabetes Association (ADA) as fasting plasma glucose (FPG) between 100 and 125 mg/dL, impaired glucose tolerance (IGT) after a 75 g oral glucose tolerance test (OGTT) of between 140 and 199 mg/dL and HbA1c of 5.7–6.4% [62]. The International Expert Committee (IEC) uses HbA1c between 6.0 and 6.4% as the only diagnostic criteria [63], whereas, according to the World Health Organization (WHO), prediabetes is defined only by FPG between 110 and 125 mg/dL and IGT between 140 and 199 mg/dL. The WHO does not support the use of HbA1c as a diagnostic criterion because it is influenced by many conditions, such as anemia, chronic kidney disease and other systemic illnesses [64]. According to a recent epidemiological evaluation, at least 1 in 3 adults in the US and 1 in 5 adults in Europe suffer from prediabetes [65].

Prediabetes is associated with a pro-inflammatory condition expressed by the elevation of different cytokines, such as tumor necrosis factor-alpha (TNF-alpha) and ICAM-1 [64], while an increased level of fibrinogen and high-sensitivity C-reactive protein (hs-CRP) is associated with a pro-thrombotic state [64].

Prediabetes evolves annually in diabetes in 5–10% of cases, and it is associated with the risk of microvascular and macrovascular complications [66]. The cardiovascular risk of prediabetes is determined by the higher prevalence of hypertension, dyslipidemia, endothelial dysfunction and an increased pro-thrombotic state. Endothelial dysfunction is more significant in prediabetic patients with IGT than impaired fasting glucose (IFG), maybe due to glucose oscillation, which causes oxidative stress and tissue damage [65]. Atherosclerosis and the subsequent genesis of atheromas are the main causes of macrovascular complications, such as coronary artery disease, myocardial infarction, stroke and peripheral vascular disease (due to arterial stiffness [64]). Recent studies are investigating the possible association between prediabetes and diastolic heart failure in humans. Microvascular complications include retinopathy, neuropathy and nephropathy [67]. According to the Diabetes Prevention Program Outcome Study (DPPOS) and others, the most successful measures for prediabetes are lifestyle modifications in order to reach a weight loss of more than 5% through both caloric restriction and physical activity. In fact, in these conditions, prediabetes may revert to normoglycemia in 19% of cases in the DPPOS [64].

Lifestyle modifications can be associated with pharmacological treatment in which semaglutide has a promising role. Semaglutide has been studied in the Semaglutide Treatment Effect in People trial with the obesity (STEP) program. This trial demonstrated that treatment with 2.4 mg of semaglutide once weekly plus lifestyle modifications in participants with prediabetes and obesity obtained better glucose control, weight loss and euglycemic conversion. Comparing semaglutide with other drugs of the same pharmacological class as liraglutide (the Satiety and Clinical Adiposity—Liraglutide Evidence in Non-diabetic and Diabetic Individuals (SCALE) trial), semaglutide was superior in achieving weight loss and normoglycemia in patients with prediabetes [68].

## 6. Semaglutide and Diabetes: When Treatment and Prevention May Come Together

As other GLP-1RA, semaglutide is recommended for the treatment of T2DM [4]. The current guidelines suggest that in recently diagnosed T2DM, treatment should start with a healthy lifestyle (avoiding excess calories and rapidly absorbed carbohydrates) and physical exercise [4,69]. If indicated, early-stage, single or combination therapy with oral glucose-lowering agents is recommended until injectable therapy with more effective drugs (insulin or GLP-1 receptor agonists) becomes necessary [4,69]. Semaglutide is the first GLP-1RA also available for oral administration, and it has been proven to be effective in reducing HbA1c, body weight and adverse events [70].

The clinical potential of semaglutide in diabetic patients has been evaluated in the “Semaglutide Unabated Sustainability in Treatment of Type 2 Diabetes” (SUSTAIN) program, including six trials wherein the primary endpoint was a change in HbA1c from the baseline to the end of the trial (ranging from 30 to 56 weeks) [71]. In addition, a cardiovascular outcome trial was performed. A total of 8416 patients with T2DM were enrolled in this program in order to evaluate semaglutide in different T2DM populations, ranging from drug-naïve to patients already treated. In the latest, semaglutide was administered in combination with metformin, thiazolidinediones, sulphonylureas, other glucose-lowering drugs (GLDs) and insulin [71]. All studies were designed as randomized controlled trials (RCTs) studying the efficacy of semaglutide versus a placebo, DPP-4 inhibitor (DPP4i), other GLP-1RAs and long-acting insulin analogs [71].

In the SUSTAIN 1 trial, two doses of semaglutide (0.5 mg and 1.0 mg once weekly) were tested vs. placebo injections in T2DM patients, plus diet and exercise for 30 weeks. In both groups, the HbA1c reduction was significantly greater than in the placebo group [72].

In the SUSTAIN 2 trial, two doses of semaglutide (0.5 mg and 1.0 mg once weekly) were evaluated vs. 100 mg of sitagliptin (DDP4) once daily for 56 weeks [73]. Most of the population enrolled was on metformin (99–100%). At the end of the study, HbA1c was significantly more reduced with semaglutide compared to sitagliptin [73].

The SUSTAIN 3 trial was the first study comparing semaglutide (1.0 mg once weekly) against another GLP-1RA (extended-release exenatide, 2.0 mg once weekly) for 56 weeks in addition to the background therapy [74]. After 56 weeks of treatment, semaglutide significantly reduced HbA1c (by 16.8 ± 0.68 mmol/mol) versus 10.0 ± 0.70 mmol/mol in the exenatide ER group [74]. The greater improvement in the glucose plasma levels of semaglutide compared to exenatide can be explained by a greater decrease in insulin resistance, measured by HOMA insulin resistance. Furthermore, the worse performance of exenatide can be linked to the more complex device system used to administer this drug. Another possible explication can be the greater formation of exenatide antibodies due to a lower homology between exenatide and native GLP-1 than semaglutide [74].

In the SUSTAIN 4 trial, semaglutide was evaluated against insulin glargine, a long-acting insulin analog. T2DM patients already treated with other GLDs (at a stable dose) were randomized to semaglutide (0.5 mg or 1.0 mg once weekly) or a starting dose of 10 IU insulin glargine [75]. After 30 weeks of treatment, HbA1c fell in all three treatments, but both semaglutide doses were significantly better than insulin glargine [75].

The SUSTAIN 5 study investigated semaglutide (0.5 or 1.0 mg once weekly) as an add-on to basal insulin therapy for 30 weeks [76]. At the end of the study, HbA1c fell significantly more with semaglutide in a dose-dependent manner compared to the placebo [76].

Another trial evaluating semaglutide against another GLP-1RA (dulaglutide) was the SUSTAIN 7 study. T2DM patients receiving semaglutide (0.5 mg or 1.0 mg) or dulaglutide (0.75 mg or 1.5 mg) were followed for 40 weeks [77]. HbA1c was significantly reduced in all four groups; however, it was significantly more with semaglutide versus dulaglutide [77]. This effect may be related to increased body weight reduction mediated by semaglutide and, consequently, a greater improvement in insulin sensitivity and beta-cell function. Furthermore, preclinical studies based on mouse animal models demonstrated that semaglutide could activate GLP-1 brain receptors due to a lower molecular weight than dulaglutide. Another important reason for the different efficacy of the two drugs may be linked to their pharmacokinetic profiles and especially to exposure over time [77].

The SUSTAIN 6 trial was designed to evaluate cardiovascular outcomes in T2DM patients on semaglutide for a follow-up of 104 weeks [78]. In line with other trials, semaglutide significantly reduced Hb1Ac, but this reduction was also associated with a lower rate of cardiovascular death, nonfatal myocardial infarction and nonfatal stroke [78].

In addition, fasting plasma glucose (FPG) and post-prandial glucose (PPG) were significantly reduced in all trials with 1.0 mg of semaglutide once weekly. Moreover, in the studies where the homoeostatic model assessment of beta-cell function (HOMA-B) and insulin resistance (HOMA-IR) were calculated, HOMA-B was found to be increased, and HOMA-IR was found to be decreased [72,73,74]. HOMA-IR was also significantly decreased compared with exenatide [74]. Furthermore, the fasting proinsulin-to-insulin ratio, which is elevated in most T2DM patients indicating a decreased insulin-secretory capacity [79], was also reduced by semaglutide, as reported in the SUSTAIN trials 1–3 [72,73,74].

Semaglutide has also been investigated with SGLT2 inhibitors. Specifically, in the SUSTAIN 8 trial, once-weekly semaglutide was evaluated versus daily canagliflozin as an add-on to metformin in T2DM patients, resulting in a superior reduction in Hb1Ac and body weight [80]. This effect was further confirmed in the SUSTAIN 9 trial, where semaglutide was added to a pre-existing antidiabetic therapy, including an SGLT-2 inhibitor in patients with inadequately controlled T2DM, resulting in a significant improvement in glycemic control and body weight reduction [81]. In addition, in uncontrolled T2DM already treated with other antidiabetic agents (from one to three drugs), semaglutide was superior to liraglutide in reducing Hb1Ac and body weight, as reported in the SUSTAIN 10 trial [82]. Finally, the SUSTAIN 11 trial evaluated semaglutide once weekly vs. thrice-daily (TID) insulin aspart (IAsp) in inadequately controlled T2DM patients already treated with insulin glargine (IGlar) and metformin, showing a better glycemic control than TID IAsp with greater weight loss [83]. A summary of the SUSTAIN program is provided in Table 2.

Based on this extensive development program, semaglutide appears to be a powerful antidiabetic agent.

## 7. Semaglutide and Cardiovascular Diseases: More than Weight Loss and Glycemic Control

The role of obesity as a significant risk factor for the development of CVD has been widely recognized [84]. It is estimated that by 2035, more than half of the world’s population will be affected by overweight or obesity, thus determining an obesity epidemic [85]. Despite significant progress in reducing cardiovascular risk by treating comorbidities, such as dyslipidemia, arterial hypertension and diabetes [86], the impact of weight reduction on cardiovascular risk remains unknown.

Semaglutide is now a reality in body weight reduction (up to 16% in conjunction with lifestyle recommendations) and glycemic control. In addition to the aforementioned effects, it may also directly act on CV risk factors, such as hyperglycemia, insulin resistance, blood pressure, dyslipidemia, waist circumference and inflammatory markers [1,87]. Thus, a reduction in CV risk has been hypothesized. The SUSTAIN 6 trial was the first study confirming noninferiority in terms of the CV safety of subcutaneous injectable semaglutide versus a placebo, determining a 26% relative reduction in major adverse cardiovascular events (CV death, nonfatal myocardial infarction, nonfatal stroke, also defined as MACE) [78]. Specifically, a significantly decreased risk of nonfatal stroke, a lower (but not significant) risk of nonfatal myocardial infarction and no difference in cardiovascular death was observed. An increase in heart rate was reported, a specific class effect of GLP-1RAs. The observed CV risk reduction might be a consequence of Hb1Ac decrease (with no higher risk of hypoglycemia versus a placebo), body weight and systolic blood pressure reduction with a modified progression of the atherosclerosis process [88]. The following PIONEER 6 study, powered to demonstrate noninferiority, showed a similar CV safety profile of oral semaglutide [89]. According to PIONEER 6, oral semaglutide reveals a CV safety profile comparable to the injectable form, as evidenced by SUSTAIN 6; thus, the pharmacokinetic properties and effects of semaglutide are not influenced by the route of administration.

Late in 2023, the SELECT study was published [12]. It is a multicenter, double-blind, randomized, placebo-controlled, event-driven, superiority trial that compares semaglutide with a placebo in addition to standard care in a population of patients who had pre-existing cardiovascular disease and a body mass index of 27 or greater but no history of diabetes. The primary cardiovascular endpoint was a composite of death from cardiovascular causes, nonfatal myocardial infarction or nonfatal stroke in a time-to-first-event analysis [12].

Patients were randomized 1:1 to receive 2.4 mg of semaglutide subcutaneously (sc) once a week or a placebo. A total of 17,604 patients underwent randomization. Eligible patients were at least 45 years old, had a BMI of 27 or greater and had established cardiovascular disease, defined by one or more of the following conditions: previous myocardial infarction (MI), previous ischemic or hemorrhagic stroke, symptomatic peripheral artery disease (PAD), defined as intermittent claudication with an ankle-brachial index of less than 0.85 (at rest), or a peripheral arterial revascularization procedure or amputation due to atherosclerotic disease. Patients were excluded if they had HbA1c > 6.5% (48 mmol/mol); a history of type 1 or 2 diabetes; had been treated with oral hypoglycemic agents or GLP1-RAs in the 90 days prior to randomization; had NYHA IV heart failure; had end-stage renal disease; or on dialysis. Patients could not be enrolled within 60 days after a cardiovascular or neurological event or if they were planning to undergo coronary, carotid or other peripheral revascularization. Patients who developed diabetes during the study remained in the study and were allowed to receive hypoglycemic drugs (excluding other GLP-1 RAs) at the discretion of the investigator.

The primary objective of the study was to demonstrate the superiority of subcutaneous semaglutide over a placebo in addition to standard therapy in reducing the incidence of MACE. The confirmatory secondary endpoints were time from randomization to the following: CV death; all-cause death; and first occurrence of a composite heart failure endpoint consisting of heart-failure hospitalization, an urgent heart-failure visit or CV death.

A primary cardiovascular endpoint occurred in 569 of the 8803 patients (6.5%) in the semaglutide group and 701 of the 8801 patients (8.0%) in the placebo group (hazard ratio, 0.80; 95% confidence interval, from 0.72 to 0.90; *p* < 0.001).

The mean age of the overall enrolled population was 61.6 years (ranging from 45 to 93 years). Of note, prediabetes (defined by HbA1c levels between 5.7 and 6.4%) was present in 64.5% of participants.

The study demonstrated that the use of semaglutide compared to a placebo reduces the composite risk of cardiovascular death, nonfatal myocardial infarction or nonfatal stroke by 20% (hazard ratio 0.80; 95% CI, 0.72 to 0.90). In addition, semaglutide resulted in a reduction in body weight (up to 9.39%) and waist circumference. The exact mechanism through which semaglutide reduces cardiovascular risk has not been fully elucidated, but several plausible hypotheses have been postulated (weight loss, an improvement in glucose homeostasis, reduction in ectopic perivascular and epicardial adipose tissue contributing to the onset of atherosclerosis and decreased systemic inflammatory and prothrombotic burden associated with obesity).

The SELECT study provides the first rigorous evidence that a pharmacological intervention for overweight or obesity can reduce the risk of cardiovascular events. This could lead to a change in the treatment paradigm for cardiovascular diseases, with greater emphasis on weight management as a strategy to reduce cardiovascular risk. The current guidelines recommend the use of GLP-1RAs in patients with diabetes who need to reduce body weight, cardiovascular risk or both [90]. The estimated global prevalence of diabetes is about 30% among patients with chronic coronary syndromes; therefore, most people with cardiovascular diseases do not have diabetes [91]. Therefore, treatment with semaglutide could be applied to a much larger population for the secondary prevention of cardiovascular events. Furthermore, about two-thirds of the patients in this study had dysglycemia (glycated hemoglobin levels from 5.7 to 6.4%); therefore, a more careful therapeutic approach to prediabetes would be necessary to improve cardiovascular outcomes through appropriate weight management.

However, the SELECT study has some limitations that deserve careful consideration. One of the main limitations is that the study included only patients with pre-existing cardiovascular disease. Therefore, the results may not be generalizable to individuals with overweight or obesity in the absence of pre-existing cardiovascular disease. Also, only 27.7% and 3.8% of the enrolled patients were women or black people, respectively; therefore, the studied population is not globally representative.

In conclusion, the results of the SELECT study suggest that semaglutide could be a promising therapeutic option to reduce cardiovascular risk in people with obesity. However, further research is needed to confirm these findings and assess the efficacy and safety of semaglutide in populations different from those studied in the SELECT study.

Finally, the semaglutide cardiovascular outcomes trial (SOUL) is an ongoing study with the aim of assessing the CV efficacy of oral semaglutide in patients affected by type 2 diabetes with established atherosclerotic cardiovascular disease (ASCVD) and/or chronic kidney disease (CKD). The results of this study will expand the evidence about the safety and efficacy of this important drug of the GLP-1RA class [92].

**Figure 1:**
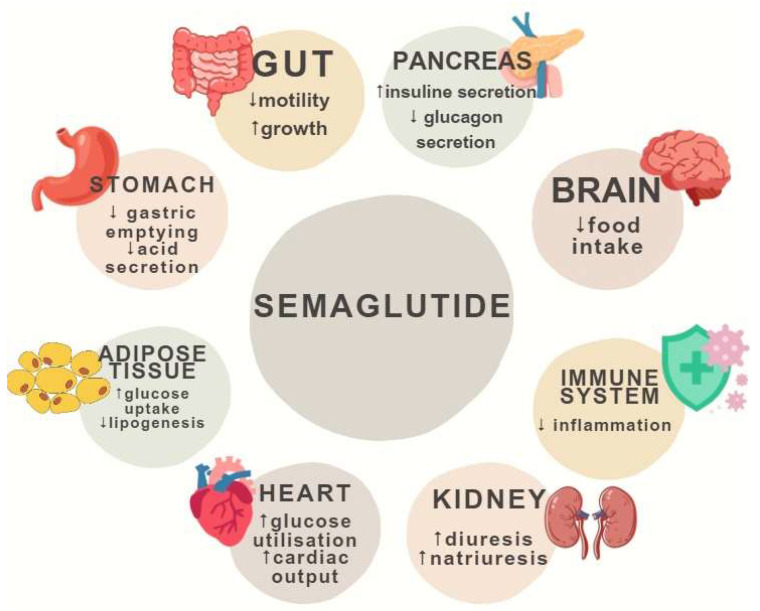
Schematic view of potential benefits of semaglutide with underlying physiological mechanisms.

A summary of the potential benefits of semaglutide is provided in Figure 1.

## 8. Discussion

The present manuscript analyzes, in detail, all the available data on the use of semaglutide in different clinical scenarios. As a GLP-1RA, its natural role is glycemic control in T2DM patients. In this regard, the drug has been evaluated in all possible combinations with other antidiabetic medications, from placebos to insulin, SGLT2i and multiple antidiabetic drug regimens. In all these conditions, semaglutide has been proven to be effective in further reducing Hb1Ac alone or as an add-on therapy [14]. However, this drug, conceived as an antidiabetic agent, is very effective in promoting weight loss (up to 10%) [44]. For this reason, an off-label use of semaglutide occurred; thus, the FDA has changed the label of this drug from “weight loss agent” to “anti-obesity medication”. There are 2 years of available data on the efficacy and safety of semaglutide. Long-term studies are still needed.

The SELECT trial further expanded the benefits of semaglutide administration. In this study, including patients with pre-existing cardiovascular disease and overweight/obesity but without diabetes, semaglutide significantly reduces the incidence of death from cardiovascular causes, nonfatal myocardial infarction or nonfatal stroke compared to the placebo. It is interesting to note that about 2/3 (up to 66%) of the population enrolled are prediabetic according to the American Diabetes Association criteria [93]. It is well known that prediabetes is a high-risk state for diabetes, and without adequate intervention, prediabetes will progress into type 2 diabetes at a rate of 5–10% per year or faster for certain ethnicities [66]. Despite a debate over when and how to intervene is still open [94], prediabetes has a prognostic role in patients with cardiovascular diseases [95], as in the population enrolled in the SELECT clinical trial [12]. In light of this, it seems that the best phenotype for semaglutide benefits is the prediabetic patient. Thus, further studies are needed to better define the role of semaglutide in the general population at cardiovascular risk without diabetes and prediabetes.

## Figures and Tables

**Table 1:** Semaglutide and obesity STEP program.

Study	Enrolled Patients	Semaglutide Dose	Vs.	Primary Endpoints	Results
**STEP 1**	1961 adults Obesity (BMI ≥ 30 kg/mq) or overweight (BMI ≥ 27 kg/mq) At least one weight-related comorbidity.	2.4 mg s.c. OW	Placebo	% change in body weight from baseline to week 68 Achievement of a reduction in body weight of 5% or more at week 68	−14.9% with semaglutide vs. 2.4% with placebo 86.4% with semaglutide vs. 31.5% with placebo
**STEP 2**	1210 adults BMI ≥ 27 kg/mq T2DM treated with either diet and exercise alone or with a stable dose of up to three glucose-lowering agents within 90 days before screening	2.4 mg s.c. OW or 1 mg s.c. OW	Placebo	% change in body weight from baseline to week 68 Achievement of a reduction in body weight of 5% or more at week 68	−9.6% with semaglutide 2.4 mg vs. −7% with 1 mg semaglutide vs. −3.4% with placebo 68.8% with 2.4 mg semaglutide vs. 57.1% with 1 mg semaglutide vs. 28.5% with placebo
**STEP 3**	611 adults Obesity (BMI ≥ 30 kg/mq) or overweight (BMI ≥ 27 kg/mq) At least one weight-related comorbidity.	2.4 mg s.c. OW	Placebo	% change in body weight from baseline to week 68 Achievement of a reduction in body weight of 5% or more at week 68	−16% with semaglutide vs. −5.7% with placebo 86.6% with semaglutide vs. 47.6% with placebo
**STEP 4**	803 adults Obesity (BMI ≥ 30 kg/mq) or overweight (BMI ≥ 27 kg/mq) At least one weight-related comorbidity	For the first 20 weeks, 2.4 mg OW plus lifestyle intervention From week 20, 2.4 mg OW	Placebo from week 20	% change in body weight from week 20 to week 68	−7.9% continuing treatment with semaglutide vs. +6.9% continuing treatment with placebo 88.7% with semaglutide vs. 47.6% with placebo
**STEP 5**	304 adults Obesity (BMI ≥ 30 kg/mq) or overweight (BMI ≥ 27 kg/mq) At least one weight-related comorbidity	2.4 mg OW	Placebo	% change in body weight from baseline to week 104 Achievement of a reduction in body weight of 5% or more at week 104	−15.2% with semaglutide vs. −2.6% with placebo 77.1% with semaglutide vs. 34.4% with placebo
**STEP 6**	401 adults (aged ≥18 years in South Korea; ≥20 years in Japan) BMI of at least 27 kg/mq with two or more weight-related comorbidities or a BMI of 35 kg/mq or more with one or more weight-related comorbidity, who had at least one self-reported unsuccessful dietary attempt to lose bodyweight With or without T2DM	2.4 mg OW or 1.7 mg OW	Placebo	% change in body weight from baseline to week 68 Achievement of a reduction in body weight of 5% or more at week 68	−13.2% with 2.4 mg semaglutide vs. −9.6% with 1.7 mg semaglutide vs. −2.1% with placebo 83% with 2.4 mg semaglutide vs. 72% with 1.7 mg semaglutide vs. 21% with placebo
**STEP 7**	375 Asian adults Overweight or obesity With or without T2DM	2.4 mg OW	Placebo	% change in body weight from baseline to week 44 Achievement of a reduction in body weight of 5% or more at week 44	−12.1% with semaglutide vs. −3.6% with placebo 85% with semaglutide vs. 31% with placebo
**STEP 8**	338 adults 1 or more self-reported unsuccessful dietary weight loss efforts BMI of 30 or greater or 27 or greater with 1 or more weight-related comorbidities	2.4 mg OW or liraglutide 3.0 mg OD	Placebo	% change in body weight from baseline to week 68 Achievement of a reduction in body weight of 5% or more at week 68	−15.8% with semaglutide vs. −6.4% with liraglutide
**STEP** **TEEN**	201 adolescents. Overweight or obesity	2.4 mg OW	Placebo	% change in body weight from baseline to week 68	−16.1% with semaglutide vs. +0.6% with placebo

OW: once weekly; OD: once daily; Semaglutide was injected subcoutaneously.

**Table 2:** Semaglutide and diabetes SUSTAIN program.

Study	Enrolled Patients	Semaglutide Dose	Vs.	Primary Endpoints	Results
**SUSTAIN 1**	388 T2DM patients HbA1c ≥ 7.0% to 10.0%	0.5 and 1.0 mg OW	Placebo	Change in HbA1c from baseline to 30 weeks	At week 30, HbA1c −1.45% with 0.5 mg semaglutide vs. −1.43% with placebo; −1.55% with 1.0 mg semaglutide vs. −1.53% with placebo; −0.02% with placebo
**SUSTAIN 2**	1231 T2DM patients HbA1c ≥ 7.0% to 10.5% eGFR > 30 mL/min/1.73 mq	0.5 and 1.0 mg OW	Sitagliptin 100 mg	Change in HbA1c from baseline to 56 weeks	At week 56, HbA1c was −1.3% with 0.5 mg semaglutide; −1.6% with 1.0 mg semaglutide and −0.5% with sitagliptin
**SUSTAIN 3**	813 T2DM patients HbA1c ≥ 7.0% to 10.5% eGFR > 30 mL/min/1.73 mq	1.0 mg OW	Exenatide ER 2.0 mg	Change in HbA1c from baseline to 56 weeks	At week 56, HbA1c −1.5% with semaglutide and −0.9% with exenatide ER
**SUSTAIN 4**	1089 T2DM patients HbA1c ≥7.0% to 10.0%	0.5 and 1.0 mg OW	IGlar	Change in HbA1c from baseline to end of treatment at 30 weeks	At week 30, 0.5 and 1.0 mg semaglutide achieved HbA1c reduction of 1.21% and 1.64%, respectively, vs. 0.83% with insulin glargine
**SUSTAIN 5**	397 T2DM patients HbA1c ≥ 7.0% to 10.0%	0.5 and 1.0 mg OW with insulin	Placebo	Change in HbA1c from baseline to 30 weeks	At week 30, HbA1c reductions with 0.5 and 1.0 mg semaglutide were 1.4% and 1.8% vs. 0.1% with placebo
**SUSTAIN 6**	3297 T2DM patients at high risk of cardiovascular events with a HbA1c level ≥ 7%	0.5 mg or 1.0 mg OW	Placebo	MACE, consisting of nonfatal myocardial infarction, nonfatal stroke or cardiovascular death at 104 weeks	The primary outcome occurred in 6.6% of patients in the semaglutide group and 8.9% of patients in the placebo group
**SUSTAIN 7**	1201 T2DM patients HbA1c ≥ 7.0–10.5%	0.5 mg or 1.0 mg OW as add-on to background treatment with metformin	0.75 mg or 1.5 mg dulaglutide as add-on to background treatment with metformin	Change in HbA1 from baseline to week 40	HbA1c was reduced by 1.5% with 0.5 mg semaglutide versus 1.1% with 0.75 mg dulaglutide and by 1.8% with 1.0 mg semaglutide versus 1.4% with 1.5 mg dulaglutide
**SUSTAIN 8**	178 adults with uncontrolled T2DM HbA1c 7.0–10.5% on a stable daily dose of metformin (≥1500 mg or maximum tolerated dose) for at least 90 days prior to screening eGFR ≥ 60 mL/min/1.73 mq	1.0 mg OW	300 mg oral canagliflozin OD	Change from baseline to week 52 in HbA1c	Change in HbA1c from baseline to week 52 of −1.5% compared with −1.0% for canagliflozin
**SUSTAIN 9**	302 T2DM adults HbA1c 7.0–10.0% despite at least 90 days of SGLT-2i	1.0 mg OW	Placebo	Change in HbA1c from baseline at week 30	At week 30, HbA1c reductions with 1.0 mg semaglutide were 1.5% vs. 0.1% with placebo
**SUSTAIN 10**	577 T2DM adults (HbA1c 7.0–11.0%) on 1–3 OAD	1.0 mg OW	1.2 mg s.c. liraglutide OD	Change in HbA1c from baseline at week 30	Mean HbA1c decreased by 1.7% with semaglutide and 1.0% with liraglutide
**SUSTAIN 11**	1748 T2DM adults inadequately controlled (HbA1c > 7.5% to ≤10.0%), with IGlar and metformin ± one additional OAD	1.0 mg OW	TID IAsp 100 U/mL	Change in HbA1c from baseline at week 52	HbA1c decreased by 1.5% and 1.2% points with semaglutide and IAsp, respectively

OW: once weekly; OD: once daily; TID: tris in die; OAD: oral antihyperglycemic drug. Semaglutide was administered subcoutaneously.

## Data Availability

The data from this manuscript are derived from publicly available published clinical trial and study results.
